# Spent Yeast-Derived 3D Porous Carbon Skeleton as Low-Cost D-Mannitol Supporting Material for Medium Temperature Thermal Energy Storage

**DOI:** 10.3390/ma16072569

**Published:** 2023-03-23

**Authors:** Xifeng Lv, Hui Cao, Guohua Li, Mengying Zhu, Wei Ji, Kai Wang, Changwei Zhang, Changsheng Su, Wenqiang Ren, Di Cai

**Affiliations:** 1 National Energy R&D Center for Biorefinery, Beijing University of Chemical Technology, Beijing 100029, China; 2 College of Chemistry and Chemical Engineering, Tarim University, Alar 843300, China; 3 Research Center for Eco-Environmental Sciences, Chinese Academy of Sciences, Beijing 100085, China

**Keywords:** yeast-derived carbon, phase change materials, medium-temperature thermal storage, D-mannitol

## Abstract

Shape-stable phase change materials (ss-PCMs) are extensively applied in renewable energy storage. The core for realizing high latent heat and good thermal stability of ss-PCMs is the designation of suitable supporting skeletons that can effectively preserve the PCMs from leaking out. In this study, ss-PCMs impregnated by D-mannitol were prepared using a waste yeast-derived carbon (YC) as the support material. YC possesses a large surface area (669.90 m^2^/g), which can provide sufficient phase transition space and nucleation sites for D-mannitol. The results indicated that a reduced supercooling of 44.76 °C for YC/D-mannitol ss-PCMs can be realized. The ss-PCMs also exhibited good cycling stability, with latent heat loss rates of 4.00% and 2.15% after 200 thermal cycles. We further demonstrate that YC provides restricted space for mannitol to inhibit the supercooling mechanism. The YC/D-mannitol ss-PCMs exhibited great promise for solar heat storage and industrial waste heat recovery in the medium temperature domain.

## 1. Introduction

Focusing on the challenges of global energy scarcity and continuously exploring sustainable ways to utilize solar energy are key to solving the energy crisis [1,2]. However, the temporal fluctuations and the unstable solar energy supplement hinder the large-scale application of the solar thermal technologies. This obstacle can be solved by adoption of thermal energy storage (TES) technologies, such as the sensible heat storage, thermochemical reactions, and the latent heat storage [3]. Among the candidate solutions, the latent heat storage (LHS) by phase change materials (PCMs) possesses high energy storage density and high latent heat, which can be maintained at almost constant temperature. Consequently, the use of PCMs has become the most popular TES technique to be investigated [4].

To date, various organic solid-liquid phase change materials including paraffin have been extensively utilized for LHS [5]. D-Mannitol is a natural sugar alcohol with advantages of high phase transition enthalpy, non-toxicity, non-corrosiveness, and low cost [6]. The solid-liquid phase transition temperatures of D-mannitol are between 165 °C and 174 °C. Therefore, it can be potentially used as a medium-temperature PCM for solar heat storage. One drawback, however, is the leakage problem, which might cause serious damage through device failure and contamination in realistic applications [7].

To solve the problem, the most effective method is the adoption of shape-stabilization phase change materials (ss-PCM), i.e., using a porous supporting matrix integrated with the organic PCMs as the working substance. In typical energy storage process by ss-PCM, the latent heat can be stored or released in the melting or solidifying processes, and the supporting matrix prevents the melted PCM from leaking out. The core to the superior TES performance by ss-PCM is the proper designation and management of the supporting material [8]. To date, various nanoparticles, polymers, and other porous materials that are rich in hierarchical pores have been explored as the PCM support (e.g., graphite [9], carbon nanotubes [10], porous aerogel [11], MOFs [12], and encapsulation by MgCO_3_ [13], and SiO_2_ [14], cellulose nanofibrils [15]); the macropores are favorable to PCM storage, the mesopores provide transport pathways, and the micropores offer capillary force.

Recently, PCM-supporting porous carbon materials that are derived from biomass have gained great interest due to their renewability and low cost. Organized hierarchical porous carbon skeletons could not only provide space for PCM encapsulation, but also provide an orderly thermal conductivity network. An et al. [16] used carbonized wood fiber as the porous support for erythritol and mannitol storage; the microporous structure of the carbon matrix enabled significant reduction of the degree of subcooling and increase of the thermal conductivity of the sugar alcohols as PCMs. In another work, Wei et al. [17] prepared a carbon aerogel from succulent plants; the abundant closed sponge-like tissues allowed a high loading rate of the organic PCM and significantly reduced liquid leakage. Liu et al. [18] prepared biomass-derived carbonaceous aerogel (BDCA)/D-mannitol ss-PCM. The porous structure in the BDCA support inhibited the supercooling of D-mannitol and improved the heat energy release.

Yeast is an ellipsoidal single-celled fungus widely found in nature. The yearly production of yeast cells and their derivatives reaches over 3 million tons/year [19]. Yeast cells are easy to cultivate, homogeneous in form, and exhibit a natural porous structure that is rich in functional groups [20]. In previous research, the spent yeast from bioethanol industry has been transformed into porous carbon by pyrolysis. Yeast-derived carbon (YC) can be used as electrocatalyst, adsorbent, and supercapacitor [21]. The stable skeleton and the unique porous structure of YC led us to consider using it as the support matrix for D-mannitol ss-PCM. In this study, for the first time, the YC/D-mannitol ss-PCM was prepared after yeast carbonization and vacuum impregnation. The phase transition behaviors and shape stability, as well as the cycling performances of the YC/D-mannitol ss-PCM, were fully characterized and investigated.

## 2. Materials and Methods

### 2.1. Materials

Wet yeast cells (*Saccharomyces cerevisiae* M 3013) were laboratory prepared by batch ethanol fermentation, according to the method described in the literature [22]. D-mannitol (98 wt%) and KOH (90 wt%) were obtained from Macklin Co., Ltd., Shanghai, China. Ethanol (98%, *v*/*v*) and H_2_O_2_ (30%, *v*/*v*) were purchased from Tianjin Fuchen Chemical Reagent Co., Ltd., Tianjin, China.

### 2.2. Carbonization of the Spent Yeast Cells

**Figure 1:**
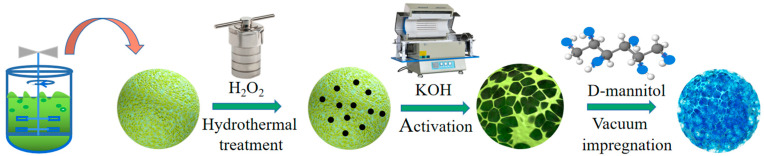
Diagram for the preparation of YC/D-mannitol ss-PCM.

The YC as the supporting matrix of ss-PCM was prepared according to the protocol showed in Figure 1. First, the spent yeast cells were collected from the ethanol fermentation broth; they were then washed 3 times with ethanol and distilled water. Then, the wet spent yeast cells were mixed with 5% (*w*/*v*) of H_2_O_2_ solution under a dosage rate of 30%, followed by hydrothermal carbonization at 160 °C for 12 h in a 50 mL Teflon-lined stainless autoclave (20 mL of working volume). The solid fraction after hydrothermal carbonization was collected and was washed with deionized water, followed by drying out at 80 °C. Finally, the solid was heated with solid KOH at a mass ratio of 1:2 at 600 °C for 4 h, with a heating rate of 5 °C /min in a N_2_ flow (50 mL/min). After being washed and drying out, the solid product was separated as the YC skeleton for ss-PCM.

### 2.3. Synthesis of the YC/D-Mannitol ss-PCM

The YC/D-mannitol was prepared through vacuum-assisted impregnation. Generally, the YC skeleton was impregnated with the molten D-mannitol in a vacuum oven at 180 °C for 4 h, followed by cooling down to room temperature (~25 °C). Subsequently, aiming to remove the excess D-mannitol, the YC after D-mannitol loading was put on a copper filter and was inserted into an oven at 180 °C. The D-mannitol adsorption capacity (*ω*) of the ss-PCM can be calculated by the following equation:

(1) $$\omega =\frac{m_{2}-m_{1}}{m_{2}}\times 100\%$$

where *m*_1_ is the weight of YC support, and *m*_2_ is the weight of YC/D-mannitol, *ω* is the molar ratio of mannitol in YC/mannitol.

### 2.4. Characterization

The surface morphology and the structure of YC/D-mannitol ss-PCM was observed by scanning electron microscopy (SEM, Hitachi, Tokyo, Japan, SU1510), and the elemental content was obtained by energy dispersive spectroscopy (EDS) in conjunction with SEM. The functional groups and chemical compatibility of the YC skeleton and YC/D-mannitol ss-PCM were evaluated by Fourier transform infrared spectroscopy (FTIR, Thermo Fisher Nicolet 6700, Waltham, MA, USA) in the wavenumber range of 4000–500/cm. X-ray diffraction (XRD) (Japan Rigaku, Tokyo, Japan, D/Max 2500 VB2 + PC) was used to analyze the crystalloid phase of the ss-PCM with a step size of 0.05° and a range from 5° to 80°. The chemical valence analysis of specimens was analyzed by X-ray photoelectron spectroscopy (XPS, Thermo Scientific K-Alpha, Waltham, MA, USA). The temperature and heat of the phase transition progress of the ss-PCM were measured by DSC from 50 °C to 180 °C in N_2_ atmosphere (Switzerland Mettler DSC 1, Greifensee, Switzerland). The heating rate was 5 °C /min. The thermal stability of the specimens was measured by TGA (Switzerland Mettler, TGA/DSC3+, Greifensee, Switzerland) for the temperature range from 30 °C to 600 °C, with 10 °C /min heating ramp in N_2_ atmosphere. The response of YC/D-mannitol to heat and the buffering effect were examined by an infrared thermography camera (FLIR T540). The specific surface area and pore size were characterized at 77 K (Micromeritics, Norcross, GA, USA, ASAP 2460) by the Brunauer–Emmett–Teller (BET) method.

## 3. Results

### 3.1. Morphology and Structure of the YC Skeleton

**Figure 2:**
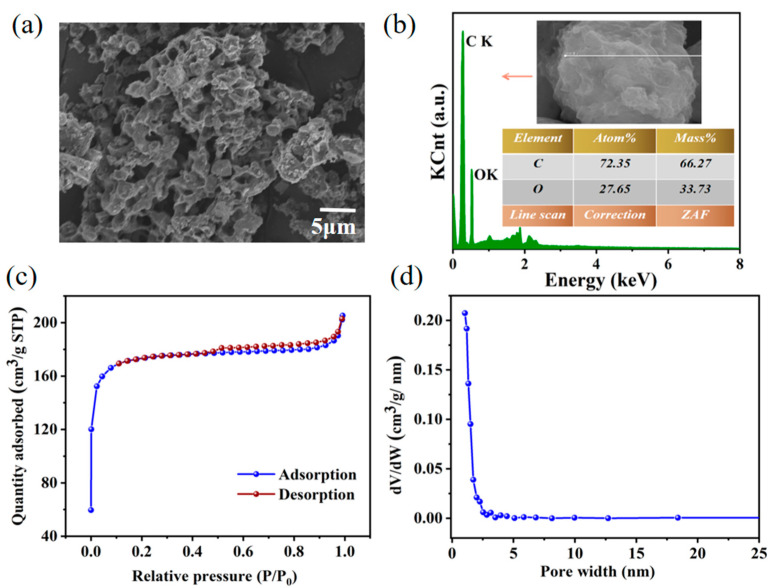
(**a**) SEM image, (**b**) EDS spectra of YC skeleton for ss-PCM. (**c**) N_2_ adsorption-desorption isotherms, (**d**) BJH pore size distribution of the YC.

Figure 2a shows the SEM image of the YC skeleton obtained in the current work. Similar to other yeast-derived carbons in the literature [21], the macropores were observed on the surface of the YC skeleton after carbonization and activation. The YC is connected by holes with different pore sizes and has a relatively fluffy structure. Therefore, we speculated that the YC exhibited a large loading space for the adsorption of PCM. The EDS energy spectrum (Figure 2b) illustrated that the YC was mainly composed of C and O elements. The carbon content of YC was 66.27% and the oxygen content was 33.73%.

Pore structure is one of the important indexes to measure the loading capacity of an ss-PCM scaffold [12]. As is shown in Figure 2c, the YC was identified as a typical IV-type isotherm with a hysteresis loop from 0.5–1 *P*/*P*_0_, confirming that the YC was rich in mesoporous structure. Additionally, isotherms of YC grew sharply at low *P*/*P*_0_ values, reflecting the microporous structure in YC. The multi-sized pore structure of the YC skeleton would clearly provide suitable spaces for the phase transition of D-mannitol when used as the ss-PCM support. In addition, the capillary effect and surface tension of the YC would also provide suitable channels to preserve D-mannitol from leakage. According to the BET and BJH models [23], the YC has a large specific surface area of 669.90 m^2^/g, and the pore volume is 0.51 cm^3^/g (Figure 2d, Table 1). We speculated the large specific surface area and suitable pore size would exhibit large adsorption capacity and strong capillary force when using YC as the support of ss-PCM.

### 3.2. Morphology and Structure of YC/D-Mannitol

**Figure 3:**
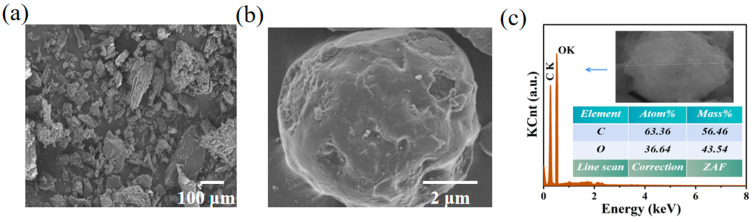
(**a**,**b**) SEM images, (**c**) EDS spectra of the YC/D-mannitol ss-PCM.

The morphology and structure of the ss-PCM using YC as the carrier material was investigated. As seen from the SEM images (Figure 3a,b), the pores on the surface of the YC were almost completely filled with D-mannitol, attributed to the storage space provided by the carbonized skeleton. As seen from the EDS spectra (Figure 3c), the O-element content of ss-PCM was 43.54% higher than that of YC (33.73%), confirming that ss-PCM could effectively be impregnated with D-mannitol. This indicates that the YC skeleton promotes the adsorption of D-mannitol and the formation of ordered thermal storage units.

**Figure 4:**
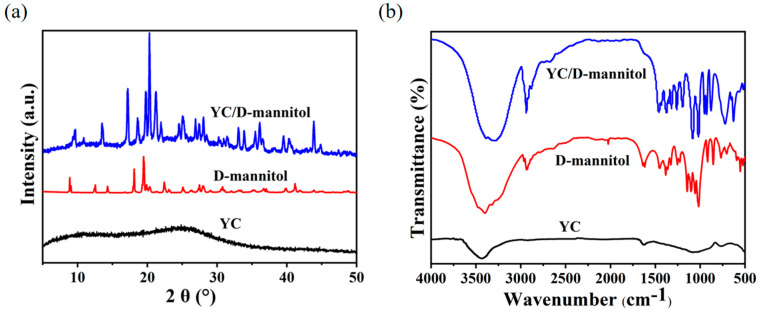
(**a**) XRD pattern, (**b**) FTIR spectrum of YC, D-mannitol, YC/D-mannitol.

In order to verify the interaction between YC support and D-mannitol, XRD diffraction of YC and ss-PCM was undertaken (Figure 4a). Two derived peaks at 25° and 43° belonged to the (002) plane and the (100) plane [24]. Therefore, the YC was rich in graphite microcrystals. The diffraction peaks were broad and weak, inferring a low degree of graphitization of the YC. The characteristic diffraction peaks of D-mannitol could be found at 2θ = 9.12°, 18.24°, 19.35° and 27.63° in the XRD patterns of YC/D-mannitol [25]. Due to a certain polymorphic transition of D-mannitol during the phase transition, a slight shift in the peak position was observed.

Figure 4b shows the FTIR spectrum of YC, D-mannitol and ss-PCM. The FTIR results implied that YC contains various functional groups; the peaks at 3455/cm and 1680/cm were the stretching vibration and bending vibration peaks of -OH [26], the peak at 1120/cm was the stretching vibration of C-O, and 730/cm was the oscillation vibration of -CH_2_ [27]. The variable angle vibration of the alcohol hydroxyl group at 1080/cm and the absorption peak of the a-type glycosidic bond at 700/cm were observed [28]. Moreover, these peaks were also detected in the spectrum of ss-PCM, indicating an effective physisorption in the YC backbone. We speculated that the intermolecular forces formed between the abundant functional groups on the surface of the YC scaffold and the -OH of D-mannitol could facilitate the adsorption of liquid D-mannitol onto YC [29]. The introduction of YC carriers significantly enhanced the absorption intensity due to the formation of heterogeneous structures and the promotion of D-mannitol crystals.

**Figure 5:**
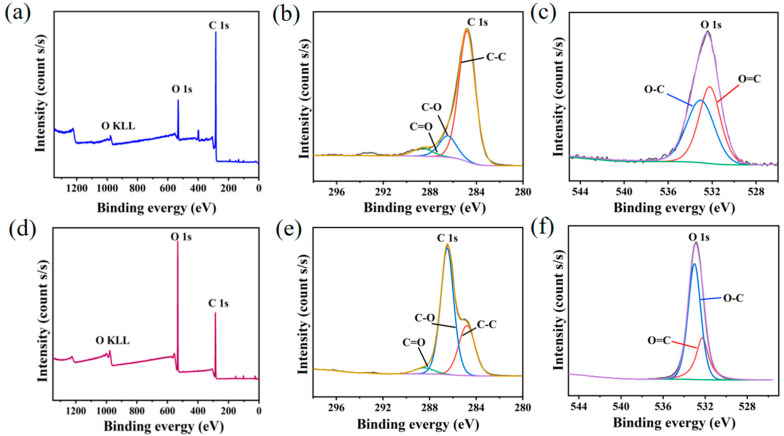
High-resolution XPS spectra of YC (**a**–**c**) and YC/D-mannitol ss-PCM (**d**–**f**).

In order to clarify the chemical state of the elements on the surface of YC before and after D-mannitol impregnation, XPS energy spectroscopy was performed. The C and O atom peaks were observed in the XPS survey spectra (Figure 5a,d) [30], which was in accordance with the EDS results. We further fitted the high-resolution XPS spectra of C 1s (Figure 5b,e) and O 1 s (Figure 5c,f). The C-C, C-O and C=O bonds were identified from the C 1s spectra at specific binding energies of 284.8 eV, 286.5 eV, and 288.5 eV, respectively. At the same time, in the O 1s spectra, the O-C and O=C bonds were determined at 533.0 eV and 532.2 eV, respectively. It is clear that the photoelectron peak of O1s in ss-PCM is significantly stronger than that of YC. Therefore, the O element content in ss-PCM is higher than that of YC. Combined with the EDS results, it can be concluded that a large amount of D-mannitol was retained in ss-PCM by vacuum impregnation.

### 3.3. The Thermal Performance of the YC/D-Mannitol ss-PCM

High supercooling was long been considered as a critical bottleneck when considering D-mannitol as a medium temperature PCM. In order to reduce the supercooling, the common strategies were adding nucleating agents, making multiple eutectics, or micro-encapsulation [31]. Nonetheless, these strategies were suffered from latent heat reduction, leakage of PCM, and high costs. Fortunately, after mixing the YC skeleton with the D-mannitol, the latent heat of phase change and supercooling can be improved, owing to the porous structure of the supporting material.

**Figure 6:**
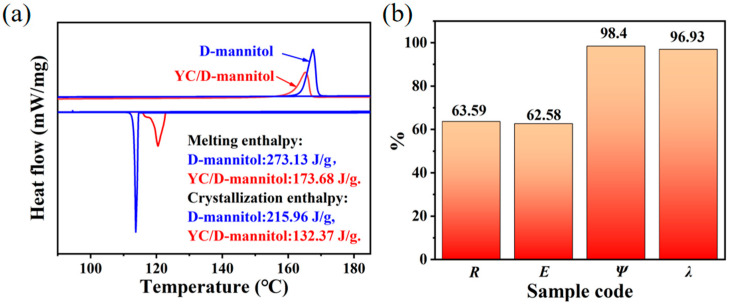
(**a**) DSC curves and (**b**) the thermal characteristics of the YC/D-mannitol ss-PCM.

In this section, the thermal performance of the YC/D-mannitol ss-PCM was further investigated. Δ*T* = *T*_m_ − *T*_c_ is the equation used to calculate the supercooling degree (Δ*T*) from melting temperature (*T*_m_) and crystallization temperature (*T*_c_). Previous research suggested D-mannitol exhibited a high degree of supercooling, which further resulted in a delaying of solidification [32]. According to the DSC curve (Figure 6a), pure D-mannitol had a *T*_m_ of 167.56 °C and *T*_c_ of 113.72 °C. Thus, the Δ*T* was as high as 53.84 °C. He [33] prepared mannitol emulsion particles to reduce the supercooling to 52.9 °C. Zeng [34] added 3% graphite to the m-Erythritol/D-mannitol binary system and the supercooling was reduced to 62.6 °C. Attractively, the YC/D-mannitol prepared in the present work possessed a supercooling of only 44.76 °C (with *T*_m_ of 165.23 °C and *T*_c_ of 120.47 °C) (Table 2), which was 16.86% lower than that of pure D-mannitol. The melting enthalpy (Δ*H*_m, ss-PCM_) and the crystallization enthalpy (Δ*H*_c, ss-PCM_) of YC/D-mannitol were 173.68 J/g and 132.37 J/g, respectively, closer to the calculated theoretical values (179.17 J/g and 141.64 J/g). Usually, owing to the inability of the carbon skeleton to phase change, a significant decrease in the molar latent heat of ss-PCM was observed. In this work, the latent heat of YC/D-mannitol of the same mass was reduced compared to pure D-mannitol. A brief comparison of the current advances of the ss-PCMs is shown in Table 3.

The attractive thermal performance of the YC/D-mannitol led us to further investigate the heat release capacity of the ss-PCM. The following equations were adopted to calculate the corresponding parameters [39].

(2) $$R=\frac{\Delta H_{m,\;\mathrm{ss}-\mathrm{PCM}}}{\Delta H_{m,\;\mathrm{Man}}}\times 100\%$$

(3) $$E=\frac{\Delta H_{m,\;\mathrm{ss}-\mathrm{PCM}}+\Delta H_{c,\;\mathrm{ss}-\mathrm{PCM}}}{\Delta H_{m,\;\mathrm{Man}}+\Delta H_{c,\;\mathrm{Man}}}\times 100\%$$

(4) $$\psi =\frac{\frac{\Delta H_{m,\;\mathrm{ss}-\mathrm{PCM}}+\Delta H_{c,\;\mathrm{ss}-\mathrm{PCM}}}{R}}{\Delta H_{m,\;\mathrm{Man}}+\Delta H_{c,\;\mathrm{Man}}}\times 100\%$$

(5) $$\lambda =\frac{\Delta H_{m,\;\mathrm{ss}-\mathrm{PCM}}}{\Delta H_{m,\;\mathrm{Man}}\times \omega_{}}\times 100\%$$

where *R* is thermal storage capacity. Δ*H*_m, ss-PCM_ and Δ*H*_c, ss-PCM_ are the melting enthalpy and crystallization enthalpy of ss-PCM, respectively. Δ*H*_m, Man_ and Δ*H*_c, Man_ are the latent heat of D-mannitol in the melting and freezing processes, respectively. *E* represents the actual percentage of stored/released energy. *Ψ* is the impregnation efficiency, and *λ* expresses relative enthalpy efficiency capacity.

As shown in Figure 6b, the *R* reached 63.59%, while the ω of YC/D-mannitol was 65.60%, indicating the effective D-mannitol impregnation in the ss-PCM; almost all of the D-mannitol molecules were involved in the solid to liquid energy conversion. The *E* value (62.58%) proved that both the heat absorption and the exothermic processes were promoted in the ss-PCM. The *Ψ* value of YC/D-mannitol is 98.40%. Almost all of the D-mannitol molecular chains were embedded in the pores of the YC skeleton, enabling the phase change during the heat absorption and exothermic release. In addition, the adsorption force and nucleation sites provided by the YC supported efficient heat storage. The high specific surface area of YC provided enough phase transition space for D-mannitol molecules. In addition, the molecular chains were almost unconstrained during the phase transition process. Therefore, the D-mannitol could easily transfer from the amorphous phase to the crystalline phase. As a result, YC/D-mannitol has a high λ value (96.93%), high crystallinity, and a lower latent heat loss. The above results demonstrate that the YC carrier not only provides a suitable backbone for the preservation of D-mannitol, but also significantly improves the thermal properties of the ss-PCM.

### 3.4. The Stability of YC/D-Mannitol ss-PCM

**Figure 7:**
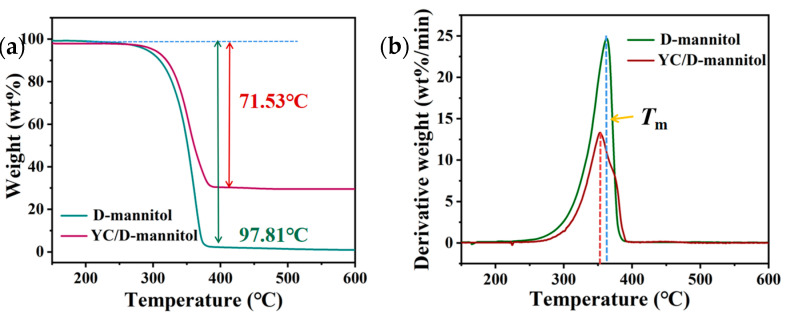
(**a**) TGA and (**b**) DTG thermograms of the pure D-mannitol and the YC/D-mannitol ss-PCM.

Considering the possible applications, the stability of YC/D-mannitol was investigated. As presented in Figure 7a, the initial thermal decomposition temperature of the pure D-mannitol was 283.5 °C, which was lower than that of the YC/D-mannitol (292.2 °C). Both pure D-mannitol and YC/D-mannitol were degraded in one step, with 97.81% of pure D-mannitol weight degradation occurring in the range of 275 °C to 375 °C. In contrast, 71.53% weight loss of YC/D-mannitol was observed between temperatures of 283 °C and 387 °C. As is shown in the DTG thermograms (Figure 7b), the *T_m__ax_* values of pure D-mannitol and YC/D-mannitol were 351.74 °C and 364.23 °C, respectively. Therefore, the introduction of YC support could significantly improve the thermally stability of the D-mannitol as PCM. This phenomenon can be attributed to the multi-level pore structure of YC, which could effectively delay the thermal decomposition of D-mannitol.

**Figure 8:**
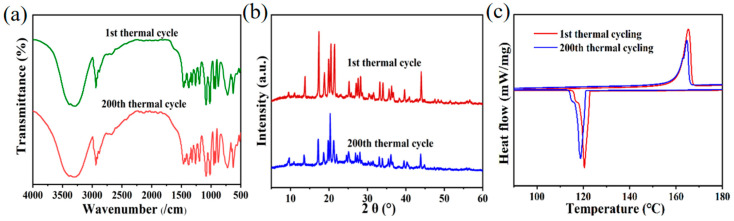
(**a**) XRD, (**b**)FTIR, and (**c**) DSC curves of the YC/D-mannitol after 200 cycles.

Long-term operation of the charging/discharging cycles of the YC/D-mannitol was conducted, in order to investigate the stability of the ss-PCM. The structure and properties of the recycled ss-PCM after 200 cycles were almost unchanged. This statement is reflected in the XRD and FTIR spectra (Figure 8a,b). As shown in Figure 8c, the DSC curves of the ss-PCM at the 1st and 200th cycles almost overlapped. Here, we may introduce the melting coefficient (*η*_m_) and crystallization coefficient (*η*_c_) to quantify the stability of the ss-PCM:

(6) $$\eta_{m}=\left|\frac{\Delta H_{m,200}-\Delta H_{m,1}}{\Delta H_{m,1}}\right|\times 100\%$$

(7) $$\eta_{c}=\left|\frac{\Delta H_{c,200}-\Delta H_{c,1}}{\Delta H_{c,1}}\right|\times 100\%$$

where Δ*H*_m,1_ and Δ*H*_m,200_ represent the enthalpy of melting. The Δ*H*_c,1_ and Δ*H*_c,200_ represent the enthalpy of crystallization in the initial state and after 200 cycles of operation.

After 200 cycles of heating charge and discharge, YC/D-mannitol had 4.00% *η*_m_ while *η*_c_ was 2.15%. The supercooling was only changed by 0.75 °C. Therefore, rapid energy release was also realized using the reused YC/D-mannitol (Table 4).

### 3.5. Leakage Analysis

**Figure 9:**
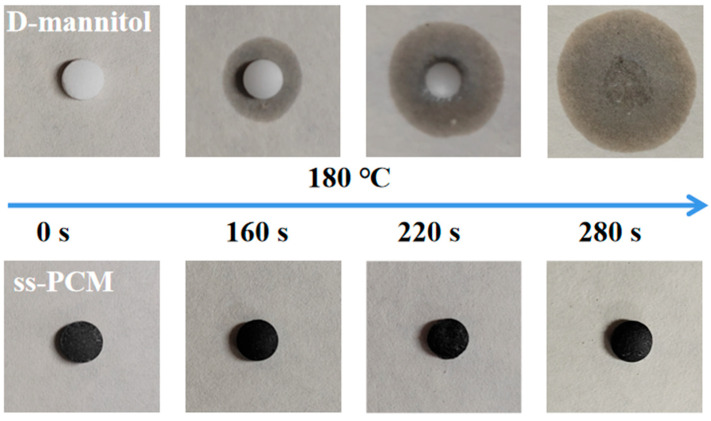
The optical images of the pure D-mannitol and the YC/D-mannitol ss-PCM on a heating stage.

Leakage analysis was further conducted. Generally, in this process, the pure D-mannitol and ss-PCM were pressed into cylinders of 0.6 mm diameter and 0.2 mm height. The cylinder specimens were placed on a heating stage at 180 °C. The state of the specimens at different times was recorded by a digital camera (Figure 9). D-Mannitol gradually changes from solid to liquid and rapidly loses its shape, completely changing to a liquid state after 280 s of heating. The ss-PCM almost kept the original shape with only a trace amount of liquid leakage during the entire heating process. This was attributed to the molecular force provided by the YC skeleton.

### 3.6. Thermal Response of the YC/D-Mannitol ss-PCM

**Figure 10:**
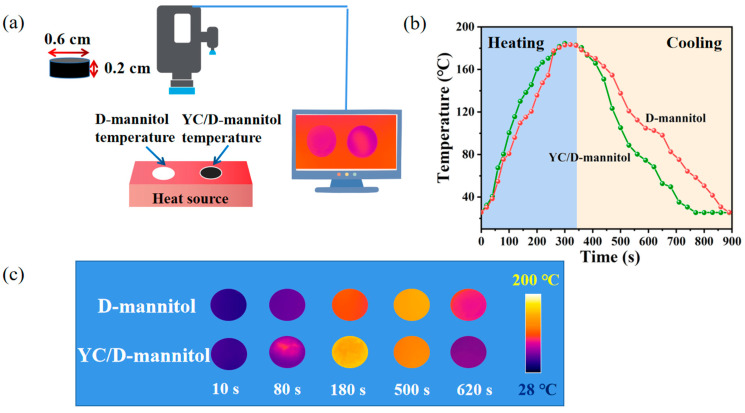
(**a**) Schematic illustration of the thermal response test, (**b**) plots of the temperature evolution, and (**c**) thermal infrared camera images of the YC/D-mannitol during the heat recovery process.

In order to visualize the thermal response behavior of the YC/D-mannitol, a homemade thermal infrared imaging platform was used (Figure 10a). During the heating process, samples were placed on the platform, and thermal infrared photographs were taken in the center of the specimens. As can be seen from Figure 10b, from the initial temperature, YC/D-mannitol and D-mannitol have to be heated for 160 s and 206 s before phase transition (the phase transition temperature was between 138 °C and 172 °C), and the phase transition temperature interval coincides with the DSC results. It took 770 s and 890 s for YC/D-mannitol and D-mannitol to cool down from the melted state to the initial state, respectively, and the heat storage and exothermic rates of YC/D-mannitol were significantly higher than those of D-mannitol. Therefore, YC could assist D-mannitol in rapid heat transfer. Through the record of the thermal infrared camera (Figure 10c), the YC/D-mannitol could be seen to exhibit good thermal responses.

## 4. Conclusions

A novel medium temperature ss-PCM was developed by vacuum impregnation of D-mannitol into the YC carrier. YC is a renewable biomass-based skeleton that was obtained by carbonizing waste yeast. The melting enthalpy and crystallization enthalpy of YC/D-mannitol reached 173.68 J/g and 132.37 J/g, respectively, and the energy storage capacity reached 98.4%. The anisotropic nucleation provided by the carrier reduced the D-mannitol supercooling to 44.76 °C. After 200 cycles, the enthalpy of crystallization of ss-PCM was only decreased by 1.8%, indicating that the YC is suitable for long-term thermal storage. This study proposed a promising way for the preparation of hierarchical porous carbonized support from the waste streams in industry, and also provided new ideas for the use of sustainable medium temperature ss-PCM.

## Figures and Tables

**Table 1:** Pore structure parameters of YC.

Sample	Specific Surface Area (m^2^/g)	Pore Volume (cm^3^/g)	Average Pore Width (nm)
YC	669.90	0.51	1.90

**Table 2:** Enthalpy and phase change temperature of the YC/D-mannitol ss-PCM.

Samples	Δ*H*_m_ (J/g)	*T*_m_ (°C)	Δ*H*_c_ (J/g)	*T*_c_ (°C)	Δ*T*
D-mannitol	273.13	167.56	215.92	113.72	53.84
YC/D-mannitol	173.68	165.23	132.37	120.47	44.76

**Table 3:** Current advances of the medium temperature ss-PCMs. The thermal performance values of the ss-PCMs are compared.

Medium Temperature ss-PCM	Δ*H*_m_ (J/g)	*T*_m_ (°C)	Refs.
D-Mannitol@Silica capsules	147.40	142.10	[25]
LiNO_3_-KCl (5/5)/expanded graphite (20 wt%)	162.00	165.58	[35]
LiNO_3_–68.3 KNO_3_	136.00	135.00	[36]
Ca (NO_3_)_2_-NaNO_3_ (3/7)/expanded graphite (7%)	89.79	216.80	[37]
KNO_3_-NaNO_2_-NaNO_3_ (53/40/7)	80.00	142.00	[38]
YC/D-mannitol	173.68	165.23	This work

**Table 4:** Thermal properties of the YC/D-mannitol before and after 200 cycles of charge and discharge.

Number of Cycles	Δ*H*_m_ (J/g)	*T*_m_ (°C)	*η*_m_ (%)	Δ*H*_c_ (J/g)	*T*_c_ (°C)	*η*_c_ (%)	Δ*T* (°C)
1st cycle	173.68	165.23	-	132.37	120.47	-	44.76
200th cycle	166.73	164.64	4.00	129.53	118.93	2.15	45.71

## Data Availability

Data are contained within the article.
